# Analytical Quality by Design: A Tool for Regulatory Flexibility and Robust Analytics

**DOI:** 10.1155/2015/868727

**Published:** 2015-02-02

**Authors:** Ramalingam Peraman, Kalva Bhadraya, Yiragamreddy Padmanabha Reddy

**Affiliations:** ^1^College of Pharmacy, Gulf Medical University, Ajman, UAE; ^2^Swaroop Tech Consultancy, Hyderabad, Andhra Pradesh, India; ^3^Analytical Research Laboratory, Raghavendra Institute of Pharmaceutical Education and Research (RIPER), Anantapur 515721, India

## Abstract

Very recently, Food and Drug Administration (FDA) has approved a few new drug applications (NDA) with regulatory flexibility for quality by design (QbD) based analytical approach. The concept of QbD applied to analytical method development is known now as AQbD (analytical quality by design). It allows the analytical method for movement within method operable design region (MODR). Unlike current methods, analytical method developed using analytical quality by design (AQbD) approach reduces the number of out-of-trend (OOT) results and out-of-specification (OOS) results due to the robustness of the method within the region. It is a current trend among pharmaceutical industry to implement analytical quality by design (AQbD) in method development process as a part of risk management, pharmaceutical development, and pharmaceutical quality system (ICH Q10). Owing to the lack explanatory reviews, this paper has been communicated to discuss different views of analytical scientists about implementation of AQbD in pharmaceutical quality system and also to correlate with product quality by design and pharmaceutical analytical technology (PAT).

## 1. Introduction

The concept of quality by design (QbD) in pharmaceutical industry has been introduced to enhance robust manufacturing process, to facilitate product quality, and to manufacture products in terms of “six sigma.” The PUCC (process of understanding the control and capability) is a loop process, implemented for continuous improvement. “Six sigma” is a system of practices developed for systematic improvement of processes, which eliminate the defects with statistical significance. Since it was originally developed, six sigma has become an important element of many total quality management (TQM) initiatives [1]. The significant number of reports on out-of-trend (OOT) results, out-of-specification (OOS) results, out-of-control (OOC) and out-of-statistical-control (OOSC), indicating that the present system of pharmaceutical industry is not immune to these issues. Hence, pharmaceutical industries are striving for new strategy and/or new element which can be add/replace the existing elements of quality and risk management system. At this juncture, implementation of QbD has been made mandatory in some countries, especially by EMA (Europe Medicines Agency) and other ICH countries [2]. International conference on hominization (ICH) Q8 (R1) guideline defines QbD as “a systematic approach to development that begins with predefined objectives and emphasizes product and process understanding and process control, based on sound science and quality risk management” [3]. It implies that product and process performance characteristics need to be scientifically designed to fulfill the specific objectives but not based on performance of test or quality control of release batches.

QbD concepts are well defined in ICH guidelines Q8 (R1): pharmaceutical development, Q9: quality risk management, and Q10: pharmaceutical quality system. There were few conferences, during late 2013 and early 2014, insisting on the implementation of the existing QbD concept to analytical method development [4, 5]. Several researchers reported that similar opportunities exist for applying QbD to analytical methods as they do for manufacturing processes [6]. AQbD helps in development of a robust and cost effective analytical method which is applicable throughout the lifecycle of the product, to facilitate the regulatory flexibility in analytical method. It means the freedom to change method parameters within a method's design space, referred to as the method operable design region (MODR) [7, 8].

Among analytical researchers, to date there is no or negligible experience or exposure with the AQbD approach for analytical methods. As today, pharmaceutical industries have many questions and require a lot more discussions on implementation AQbD and its correlation with other components of pharmaceutical quality systems. Literature survey reveals that many researchers have adopted QbD principles to the development of analytical methods and they are termed analytical QbD (AQbD) [9–25]. Certainly, most of works were not enough to define the way of implementation of AQbD, because people felt that implementation of DoE in analytical method is QbD and it is incorrect. Moreover these reports have reflected the inadequacy of knowledge on analytical target profile (ATP), method performance characteristics, risk assessment, choice of DOE tool in QbD process, optimization of MODR region and its verifications, and so forth.

We know that system suitability testing (SST) for an analytical method is required by USP and FDA to ensure ongoing performance of an analytical system and relevant methods. Very recently related chapters have been updated by United States Pharmacopoeia (USP-NF) and European Pharmacopoeia (EP) in which flexibility is granted for an analytical method that can be changed without the need for revalidation if AQbD approach has been implemented. The USP chapter 〈1058〉 makes a statement that SST can substitute an instrument's performance qualification; however further guidelines are not given. So there are many questions which still exist among regulatory expertise, and thus the concept of QbD in analytical method development (to be termed as AQbD) became a continuing interest to discuss and to learn more.

## 2. Regulatory Perspective of AQbD

With reference to pharmaceutical quality system (ICH Q 10 guidelines), analytical methods are key part of control strategy. Thus implementation of analytical QbD in manufacturing process as control strategy will ensure predetermined performance and product quality. This includes parameters and attributes related to drug substance and drug product materials components including facility, instrument operating conditions, finished product specification, and the associated methods and frequency.

Implementation of AQbD is expected to strengthen the concept of “right analytics at right time” which plays significant role in drug product development cycle. Few months ago, FDA has approved a few new drug applications based on analytical QbD and referred the importance and benefits of QbD in analytical method development [26]. It triggers the role of analytics in the product development cycle for understanding drug excipient interactions and for the measure of critical quality attributes (CQA) during experiment, process, control, and also continuous process verification in order to monitor trends in the product quality. So this resonance has attracted the pharmaceutical industry to evoke the concept of analytical quality by design (AQbD). Though cGMP regulations have been in place in the past one decade, the significant number of QC related warning letters issued by FDA demonstrated that companies have problems with risk management system in analytical methods and related systems. Quality assurance personnel believes that AQbD will be a better solution to avoid OOT and OOS and to reduce risk in method failure.

Due to the above cited discussion, analytical method development using QbD approach is a current area of focus and needs to be implemented. The process of developing and validating analytical methods parallel to product QbD benefits the quality of the product furthermore, with high degree of assurance, and it may be similarly benefitted. The dependence of pharmaceutical development and manufacture on robust analytical data intensifies the need for rigor in analytical method development and increasingly an analytical QbD (AQbD). ICH Q8 (R2) guidelines do not discuss analytical method development in correlation with design space; however it is understood that the concept can be applied to analytical design space and continuous improvement in method robustness and understanding [27]. In fact analytical methods are the indicator of quality of process, product, and robustness throughout the life cycle.

## 3. OFAT versus AQbD in Analytical Method Development

In present days, analytical method failure is becoming more common especially during method transfer as well as in quality control departments. It is presumed to be due to the exception given for robust test compliance by ICH Q2 guidelines. In current practices, chromatographic methods are more commonly employed as right analytics at all the stages during the product life cycle. Common analytical methods for content uniformity, assay, impurity profile, and stability indicating assay are based on high performance liquid chromatographic (HPLC) or ultraperformance liquid chromatographic (UPLC) or rapid resolution liquid chromatographic methods (RRLC). In connection with chromatography, due to complex parameters involved in the method development phase, low sensitivity, selectivity, and inadequate understanding between method performance and method parameters, always the revalidation protocol has been recommended in procedures. On the other hand, in current practice, the implemented analytical methods were based on one factor at a time (OFAT), in which one parameter alone is optimized for the expected response whilst others remained constant [28]. This practice always yielded a narrow robust behavior of the method for instrumental variables used in method development phase. Hence the present strategy of analytical method (i.e., OFAT) development has high risk in method failure and always requires revalidation protocol after method transfer or alternative method development; thereby it has been increasing the cost of the method.

The AQbD explores scientific understanding in method implementation sequences and starts with product quality that relates the risk assessment in method choice and then between method parameter and expected method results and finally a region for high robust and cost effective approach. DoE is a part of AQbD, and it represents the interaction among the input variables that ultimately affect the method response and results. At this juncture, AQbD paradigm is a preferred and recommended strategy to be followed in analytical method development so as to attain regulatory flexibility and reduce OOS, OOT, OOC, and OOSC results, high degree of robustness and cost effective analytical method.

## 4. Implementation of AQbD

The application of QbD concept to analytical method is justifiable, because of many variables that significantly affect the method results. These variables are such as instrument settings, sample characteristics, method parameters, and choice of calibration models. Being chromatographic technique is the most common analytical tool in pharmaceutical quality control, and the number of variables involved in analytical method development phase is almost equivalent to the number of variables involved in formulation and development protocols for dosage form. As per FDA, analytical techniques and methods play an essential role in QbD paradigm, and real time release testing and nontraditional testing techniques provide valuable information for in-process control and improvement. Implementation of QbD provides an opportunity to achieve regulatory flexibility but requires high degree of robustness, product quality, and analytical method understanding. To adopt a suitable design of experiments (DOEs) protocol in AQbD approach to identify a validated MODR for high degree of process-product-analytical method understanding is recommended [26]. Implementation QbD in analytics is parallel process to that of product QbD and is shown in Table 1. The stagewise implementation AQbD paradigm in pharmaceutical quality system is presented in Table 2.

Initially, implementation AQbD depends on the target measurement which comprises the product file in the form of ATP (analytical target profile) and CQA (ATP is the analogue of QTPP in product design), followed by an understanding on selection of suitable analytical technique, risk assessment for variables, method development using DoE, and validation process for model and control strategy. At final stage AQbD focuses on life cycle management which includes control strategy and continual improvement.

### 4.1. Analytical Target Profile (ATP)

AQbD is started with an analytical target profile or ATP, which is an analogue to QTPP (Table 1). ATP defines the goal of the analytical method development process, relating the results of the method to achieve QTPP. Recently PhRMA and EFPIA provided the definition of ATP: “ATP is a statement that defines the method's purpose which is used to drive method selection, design, and development activities.” ATP is a key parameter in AQbD that facilitates greater continuous improvement of analytical methods and their choice, once the regulatory authorities approve the ATP statement. In pharmaceutical industry, internal change control management system is responsible for effective implementation of ATP to provide regulatory flexibility [29, 30] (Table 3).

### 4.2. Analytical Method Performance Characteristics

Analytical method performance characteristics are defined to meet the need of analytical target profile. Various method performance characteristics are given in Table 4. There are two types of method performance, that is, systematic (bias) and inherent random (variance) components. In general method performance is not assessed by one but depends on both. There are many validation parameters that have been listed by USP and ICH for chromatographic separations, which are considered as method performance characteristics. Among these parameters, accuracy and precision are quite commonly considered as method performance characteristics to quantify the substance. It is assumed that no method can be accurate and precise without adequate specificity, linearity, and peak resolution. However, these performance characteristics do not represent robust behavior of the method. Range is also an important component that one has to be establish based on acceptable behavior of both systematic and random performance characteristics. Robustness defines an operational range of method factors to give defined results. But both range and robustness are neither categorized as systematic variability nor categorized as random variability. Hence, it is always recommended to incorporate a joint criterion of two or more method performance characteristics in ATP. Other method performance characteristics such as linearity and specificity are not needed to be incorporated in the ATP, as they are not directly linked to understand the agreement of a measurement with the true value. For example, an assay ATP should include a statement of accuracy and precision but not necessarily include linearity and specificity [31, 32].

### 4.3. Selection of Analytical Techniques

This must be done with reference to the needs, which are defined in the ATP. In other side, the selected analytical technique should satisfy the required method validation parameters as required by regulatory requirement. For example, specificity may not be included in ATP, but the analytical technique should satisfy the specificity. Hence chromatographic method can satisfy the required method performance defined in ATP and validation requirement of  ICH. Instead the UV spectrophotometric method can fulfill the needs of ATP but may not satisfy ICH Q2 [33].

### 4.4. Risk Assessment

Risk assessment identifies the critical method variables, the parameters that impact the ATP. Once the technique is identified, AQbD focuses on method development and includes detailed assessment of the risks associated with variability such as analyst methods, instrument configuration, measurement and method parameters, sample characteristics, sample preparation, and environmental conditions. Risk assessment strategy needs to fulfill the ICH Q9 guideline: “it is systematic process for the assessment, control, communication and review of risks to the quality across the product lifecycle” [34]. It is done throughout the life cycle and typically performed at the end of the method development stage. It is a precursor to method transfer and focus on routine laboratory practices, reagent sources, testing period, and cycle time. Hence it is recommended to notify the major differences during the technique selection and its development stage. For example, in chromatographic separation, the variability due to HPLC instrument configuration, column selection, flow rate, injection volumes, and so forth are kept controlled in the experimental strategy, while rest of the variables (pH, column temperature, and % ration of mobile phase) can be assigned to test the robust behavior and for the establishment of MODR. A design of experiments (DOE) approach is adopted to define the MODR. As a part of pharmaceutical quality system, an analytical method failure and risk factor are correlated with the following:

(1) $$\begin{array}{c} \text{Risk}\;\text{factor}=\text{Severity}\times \text{Occurrence}\times \text{Detectability}, \end{array}$$

where Severity is effect on patient related to safety of efficacy (CQAs), Occurrence is chance of failure related to product, process knowledge, and control, and Detectability is ability to detect a failure capability of analytical method and sampling.

### 4.5. Design of  Experiments

In accordance with the requirement of ICH Q8 guidelines, regarding “design space” in product development, method operable design region (MODR) can also be established in method development phase, which could serve as a source for robust and cost effective method. MODR is the operating range for the critical method input variable (similar to CQAs) that produces results which consistently meet the goals set out in the ATP. MODR permits the flexibility in various input method parameters to provide the expected method performance criteria and method response without resubmission to FDA. It is based on a science, risk based and multivariate approach to evaluate effects of various factors on method performance. FDA has suggested conducting MODR together with method validation as most recommended. Once this is defined, appropriate method controls can be put in place and method validation can be carried out. There are many analytical works which have been reported using experimental design based on factorial or fractional factorial design or response surface methodology. But those works were limited to the development of mathematical models to correlate input variables (*X*_*n*_) and output responses (*Y*_*n*_). The implementation of DoE in method development phase requires a huge understanding in selection of input variables and output response. DoE in AQbD approach includes the following.

#### 4.5.1. Screening

In screening, qualitative input variables can be screened out. It identifies the various critical method parameters (CMP) to be considered in the optimization experiments. In addition, it also works as a semioptimization tool to indicate the required levels of CMA for an optimization experiments. The various tool and selection approaches are shown in Table 5. The screening experiments should conclude the segregation of CMP that need to be either controlled or subjected to DOE techniques in MODR optimization.

#### 4.5.2. Optimization

In this stage, quantitative measures for critical method in variables (i.e., CMP) either from screening or directly from risk assessment can be incorporated. It provides a base for scientific understanding of relation between quantities of input variables (CMP) and output response which will show considerable effect on method performance and ATP.

#### 4.5.3. Selection of DOE Tools

During the optimization, many approaches can be used to derive a mathematical relationship (model). The decision on selection of tool for DoE has to be made based on the number of input variables, knowledge on controlled parameters, and scientific understanding between result and variable (if any). Statistical knowledge is prime importance to interpret the interaction and contribution of variables (*X*_*n*_) in method responses (*Y*_*n*_), serving as a tool to select the variables at optimum levels. For example, if the effect of all input variables and their interactions are to be measured, factorial design can be applied then it can be considered and optimized with RSM (response surface methodology). Taguchi method can be used with lower number of experimental runs compared to factorial designs (say, 50%, 25%, etc.) but the interactions confounded need to be resolved. Where large numbers of input variables are to be studied without interaction effects, Plackett-Burman methods can be used. A typical selection of techniques is shown in Table 5.

#### 4.5.4. Method Operable Design Region (MODR) and Surface Plots

**Figure 1:**
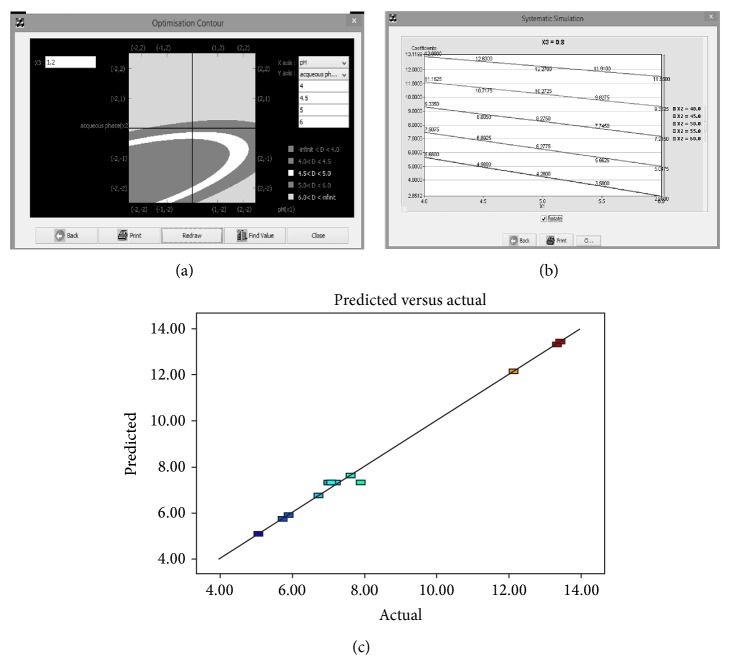
(a) Contour plot for MODR (retention time as method response). The above graph shows the different shade for different region for retention time at different levels −2, −1, 0, +1, and +2. (b) Systematic simulation graph for retention time (*y*-axis) as method response at constant X3 (0.8 mL/min as flow rate) with change in pH (X1-*x*-axis). (c) Graph shows significant correlation between the predicted retention time and actual (experimental) retention time with good correlation coefficient.

A model contour plot (2D plot) for MODR concept is shown in Figure 1(a). The contour plot is a 2D response plot representing the impact of pH (*x* axis) and % aqueous phase (*y*-axis) on retention time of analyte, whilst factors like flow rate and other instrument configurations are controlled. Numbers like −1, −2, +1, and +2 in both axes represent the coded level of variables used in DOE plan.

This contour is suitable for the response if it is nonlinear and the relationship between input variable and method response is having more curvature effect. Then MODR can be selected from contours using mathematic models. The predicted value of method response can be verified by using actual experimental run as a part of model validation. There is another surface model that can be obtained by means of simulation that provides the change of response with respect to variables, which is more suitable for linear relationship (Figure 1(b)).

#### 4.5.5. Model Validation

Prior to the choice from contour or graph, the predicted values for the targeted method response have to be validated by actual experimental run. Then the regression analysis has to be carried out to validate the model statistically. The representative graph has been shown in Figure 1(c).

## 5. Method Verification/Validation 

In general validation of an analytical method is always done as per ICH Q2 (R1) guidelines under normal operating condition (NOC) or the so-called optimized condition comprising set of variables at one point. In addition to method validation as per regulatory guidance, method verification can be performed through a joint accuracy and precision assessment at different method factor points within the chromatographic separation space (from MODR). This multipoint verification within MODR likely represents greatest probability on method's ability to meet the requirement of ATP. This multipoint verification should be done beyond the regular robust test limits at more than two points with both deviations but see that points are from the MODR. For example, column temperature can be verified between 35°C and 45°C. The percentage aqueous or organic components in mobile phase may be verified up to ±5%, and pH can be ±0.3 unit. The validation and verification experiments should demonstrate robust across the parameter ranges from low to high through a target value of variable. For example, if target buffer concentration is 15 mM, the robust check has to be conducted from 10 mM (low) to 20 mM (high). If the performance characteristics are satisfied and meet the ATP, then method operable range for buffer concentration can be 10–20 mM, provided that specificity is being proven.

## 6. Control Strategy/Conformance to ATP

In product QbD, control strategy is designed to ensure the instant product production with required quality. Control strategy is derived from various data collected during method development phase and method verification process. This data correlation will predict the ability of method to meet ATP criteria and control strategy, including the overall monitor of method parameters that significantly influence method (variability). It is noted that method control strategy of AQbD approach does not differ from the traditional control strategy. However, method controls need to be established to ensure relation between method purpose and method performance [34].

## 7. Continuous Monitoring/Lifecycle Management

This follows the establishment of an analytical method for quality control or routine testing and is established by monitoring the method performance over the time to ensure that the method remains in compliance with the defined ATP criteria. In pharmaceutical industry, it is represented by using control charts or other tools to track system suitability data and method related investigations. This continuous monitoring allows an analyst to detect, identify, and address any abnormal or out-of-trend performance of the analytical method.

The role of analytical method in control strategy is critical, and it begins from raw material testing (before manufacturing) to stability testing's (after marketing), as shown in Table 6.

## 8. PAT and AQbD

For the effective implementation of process analytical technology (PAT) system, parallel development of analytical QbD is highly recommended. PAT is based on two major components: (a) understanding of the scientific and engineering principles involved manufacturing process; (b) identification of the variables which affect product quality. According to the FDA draft guidance, “the desired state of pharmaceutical manufacturing is that product quality and performance are ensured through the design of effective and efficient manufacturing processes” in which continuous and real time quality assurance was recommended. Once the properties of the drug product components are understood, the processing variables that control the relevant properties must be identified. Identification of these variables necessarily requires a multivariate approach. Now, pharmaceutical industries are in progress of establishing specific process understanding and design process analytical control strategies to make PAT approach more effective tool [34].

## 9. How to Implement in the Current Practice

In future, analytical QbD has to be introduced in the method development phase and has to be validated for the method performance along with the validation protocol. For a given drug product, to implement analytical QbD, the following may be considered.

- Construct a QTPP (a profile based on the product specification as outlined in FDA approval).
- Analyze each product specification for criticality.
- Assess and justify the analytical method development and its suitability to support the criticality.
- Select the suitable analytical method like HPLC, UV, and IR to meet ATP and then QTPP.
- Perform risk assessment for selected method.
- Identify the quantitative and qualitative variable that affects the method performance and method responses to be measured.
- Use suitable DOE experiment to optimize variable and establish scientific understanding.
- Find the region, models to assess the robust, and economic operation for the method variable.
- Validate the models and MODR region using experimental verification at different points to prove robustness.
- Then validate the method in the operable region for the method performance and subject to control strategy and improvement.

## 10. Regulatory Considerations for Current and Future

The FDA and EMA recently announced a joint collaboration that began in January 2013. The goals of the collaboration are to (1) develop analytical methods (e.g., HPLC) based on the QbD paradigm; (2) define protocols for method transfer; (3) establish methodology for verification of the MODR upon site transfer; and (4) define review criteria for evaluation of QbD based analytical methods.

## 11. Recommendations

Analytical methods for existing drugs have to be periodically reevaluated to address any gaps or any improvement in method performance, risk factors using AQbD. Range of robustness for the method needs to be proven by verification and scientific understanding in order to avoid method failure in method transfer and also to reduce OOS and OOT.

## Figures and Tables

**Table 1:** Regulatory perspective; product QbD versus analytical QbD.

Stage	Product QbD	Analytical QbD
Stage 1	Define quality target product profile (QTPP)	Define analytical target profile (ATP)
Stage 2	Critical quality attributes	Critical quality attributes
Stage 3	Risk assessment	Risk assessment
Stage 4	Design space	Method operable design region
Stage 5	Control strategy	Control strategy
Stage 6	Life cycle management	Life cycle management

**Table 2:** Conventional approach versus product development QbD versus analytical QbD.

Parameter	Traditional	Product QbD	AQbD
Approach	Based on empirical approach	Based on systematic approach	Based on systematic approach

Quality	Quality is assured by end product testing	Quality is built in the product and process by design and scientific approach	Robustness and reproducibility of the method built in method development stage

FDA submission	Including only data for submission	Submission with product knowledge and process understanding	Submission with product knowledge and assuring by analytical target profile

Specifications	Specifications are based on batch history	Specifications are based on product performance requirements	Based on method performance to ATP criteria

Process	Process is frozen and discourages changes	Flexible process with design space allows continuous improvement	Method flexibility with MODR and allowing continuous improvement

Targeted response	Focusing on reproducibility, ignoring variation	Focusing on robustness which understands control variation	Focus on robust and cost effective method

Advantage	Limited and simple	It is expended process analytical technology (PAT) tool that replaces the need for end product testing	Replacing the need of revalidation and minimizing OOT and OOS

**Table 3:** Implementation of analytical QbD in pharmaceutical quality system.

S. number	Implementation stagewise	Description
1	Target measurement	Determine what to measure and where/when to measure it. Define ATP and develop measurement requirements based on product QTPP and CQA.

2	Select technique	Select appropriate analytical technique for desired measurement defined in ATP. Define method performance criteria.

3	Risk assessment	Assess risks associated with method input variables, sample variation, and environmental conditions. Risk assessment tools (e.g., FMEA) can be used.

4	Method development and validation	Examine potential multivariate interactions by DoE and define MODR to understand method robustness and ruggedness.

5	Control strategy	Define control space and system suitability; meet method performance criteria to meet ATP.

6	Continual improvement	Monitor method performance that remains compliant with ATP criteria and thus analysts proactively identify and address the out-of-trend performance of the method. Update with new process and analytical technology.

**Table 4:** Type of method performance characteristics as per USP and ICH Q2 (R1).

S. number	Method performance characteristics	Defined by ICH and USP
1	Accuracy, specificity, and linearity	Systematic variability
2	Precision, detection limit, and quantification limit	Inherent random variability
3	Range and robustness	Not applicable

**Table 5:** Selection of DOE tools in analytical quality by design.

Design	Number of variables and usage	Advantage	Disadvantage
Full factorial design	Optimization/2–5 variables	Identifying the main and interaction effect without any confounding	Experimental runs increase with increase in number of variables

Fractional factorial design or Taguchi methods	Optimization/and screening variables	Requiring lower number of experimental runs	**Resolving confounding effects of interactions is a difficult job**

Plackett-Burman method	Screening/or identifying vital few factors from large number of variables	Requiring very few runs for large number of variables	It does not reveal interaction effect

Pseudo-Monte Carlo sampling (pseudorandom sampling) method	Quantitative risk analysis/optimization	Behavior and changes to the model can be investigated with great ease and speed. This is preferred where exact calculation is possible	For nonconvex design spaces, this method of sampling can be more difficult to employ. Random numbers that can be produced from a random number generating algorithm

Full factorial design	Optimization/2–5 variables	Identifying the main and interaction effect without any confounding	Experimental runs increase with increase in number of variables

**Table 6:** Role of analytical method in pharmaceutical testing and control strategy.

S. number	Pharmaceutical testing	Control strategy
1	Raw material testing	Specification based on product QTPP and CQA Effects of variability, including supplier variations, on process and method development are understood

2	In-process testing	Real time (at-, on-, or in-line) measurements Active control of process to minimize product variation Criteria based on multivariate process understanding

3	Release testing	Quality attributes predictable from process inputs (design space) Specification is only part of the quality control strategy Specification based on patient needs (quality, safety, efficacy, and performance)

4	Stability testing	Predictive models at release minimize stability failures Specification set on desired product performance with time
